# Outcomes of pregnancies complicated by cirrhosis: a retrospective cohort study

**DOI:** 10.1186/s12884-024-06341-1

**Published:** 2024-03-07

**Authors:** Zhangmin Tan, Peizhen Zhang, Jin Zhou, Chuo Li, Chengfang Xu, Yuzhu Yin

**Affiliations:** Departments of Obstetrics and Gynecology, The Third Affiliated Hospital of Sun Yat-Sen University, No. 600 Tianhe Road, Guangzhou, 510630 China

**Keywords:** Liver cirrhosis, Logistic models, Obstetric outcome, Pregnancy, Preterm birth, Prognosis

## Abstract

**Background:**

Although pregnancy complicated by liver cirrhosis is rare, women with cirrhosis experience increased adverse pregnancy outcomes. This study aimed to evaluate pregnancy outcomes in women with liver cirrhosis and develop a predictive model using maternal factors for preterm birth in such pregnancies.

**Methods:**

A retrospective analysis was conducted on pregnancy outcomes of a cirrhosis group (*n* = 43) and a non-cirrhosis group (*n* = 172) in a university hospital between 2010 and 2022. Logistic regression evaluated pregnancy outcomes, and a forward stepwise logistic regression model was designed to predict preterm birth in pregnant women with cirrhosis. The model's predictive performance was evaluated using the receiver operating characteristic (ROC) curve and the area under the ROC curve (AUC).

**Results:**

The incidence of cirrhosis during pregnancy was 0.06% (50/81,554). Pregnant women with cirrhosis faced increased risks of cesarean section, preterm birth, intrahepatic cholestasis of pregnancy, thrombocytopenia, and postpartum hemorrhage. In pregnant women with cirrhosis, preterm birth risk significantly increased at an incidence rate of 46.51% (20/43). According to the prediction model, the key predictors of preterm birth in pregnant women with cirrhosis were intrahepatic cholestasis of pregnancy and total bilirubin. The model demonstrated accurate prediction, with an AUC of 0.847, yielding a model accuracy of 81.4%.

**Conclusions:**

Pregnant women with cirrhosis face a heightened risk of adverse obstetric outcomes, particularly an increased incidence of preterm birth. The preliminary evidence shows that the regression model established in our study can use the identified key predictors to predict preterm birth in pregnant women with cirrhosis, with high accuracy.

## Introduction

Pregnancy complicated by liver cirrhosis is rare in clinical practice; the incidence of cirrhosis in women of childbearing age is approximately 45 per 100,000 [1]. Complications associated with liver cirrhosis include ascites, spontaneous bacterial peritonitis, encephalopathy, hepatorenal syndrome, and bleeding from esophageal varices, etc. [2]. In addition to these specific complications of cirrhosis, pregnant women with cirrhosis are at increased risk of complications during pregnancy, such as miscarriage, preterm birth, higher rates of cesarean section, and maternal death [3, 4]. Compared to pregnant women without cirrhosis, those with cirrhosis are at increased risk of adverse pregnancy outcomes. The interaction between cirrhosis and pregnancy is bidirectional. Pregnancy can lead to thrombocytopenia, decreased albumin levels, and increased portal hypertension due to an elevated total blood volume, which may further exacerbate cirrhosis. Liver cirrhosis also increases the risk of adverse pregnancy outcomes and complications [5–7].

Previous studies from Canada, Sweden, and the United States have reported associations between cirrhosis and other adverse maternal and infant outcomes [8–10]. These studies have indicated that alcoholic liver disease or nonalcoholic fatty liver disease is the main cause of cirrhosis in pregnant women. The etiology of liver cirrhosis varies according to the economic status of countries. However, there is a lack of data on pregnancy in women with cirrhosis in China.

The American College of Gastroenterology (ACG) Clinical Guideline: Liver disease and pregnancy was published in 2016 [11], while the European Association for the Study of the Liver (EASL) Clinical Practice Guidelines on the management of liver diseases in pregnancy were published in 2023 [12]. However, these guidelines do not specifically address the management of pregnant women with cirrhosis. There are currently no guidelines available for diagnosing and treating pregnant women with cirrhosis.

Management of cirrhosis during pregnancy remains a major challenge. Pregnant women with cirrhosis are at considerably higher risk of preterm birth. Furthermore, a prediction model that can accurately predict the risk of preterm birth in pregnancies complicated by liver cirrhosis has not been developed yet. The purpose of our study was to describe the maternal and fetal outcomes of pregnant women with cirrhosis, identify adverse effects of pregnancy with cirrhosis in mothers and neonates, and establish a logistic regression equation to predict the occurrence of preterm birth among pregnant women with cirrhosis.

## Methods

### Study design

A retrospective analysis was conducted on 81,554 pregnant women who gave birth at the Third Affiliated Hospital of Sun Yat-sen University between 2010 and 2022. Women with cirrhosis were initially identified according to the *International Classification of Diseases*, 10th Revision (ICD-10) [13]. A computer search of these women aimed to identify cases with liver cirrhosis (ICD-10 O26.604, pregnancy with cirrhosis; ICD-10 K74.100, liver cirrhosis; ICD-10 K74.607, decompensated cirrhosis; ICD-10 K74.3, primary biliary cirrhosis; ICD-10 K71.7, drug-induced cirrhosis; ICD-10 K70.3, alcoholic cirrhosis; ICD-10 K74.6, other causes of cirrhosis). The ICD-10 code for cirrhosis before or during pregnancy, the ICD-10 code for hepatic decompensation, and the ICD-10 code for causes of cirrhosis were applied to reduce the potential for missing a case of liver cirrhosis. In addition, each case that was considered to be liver cirrhosis was reviewed by two authors to reduce the number of false-positive cases. We identified 50 cases of pregnant women with cirrhosis. The inclusion criteria for this study were patients aged ≥ 18 years with a gestational age ≥ 28 weeks at the time of giving birth. Women with a previous history of liver transplantation were excluded from the study. Out of the 50 pregnant women with cirrhosis, seven chose to terminate their pregnancies before 28 weeks due to reasons such as decompensated cirrhosis, thrombocytopenia, and coagulopathy. None of these women had spontaneous abortions. The remaining 43 pregnant women met the inclusion criteria and were assigned to the cirrhosis group. All 43 pregnant women had been diagnosed with cirrhosis prior to pregnancy. Additionally, 172 pregnant women without cirrhosis were randomly selected from the non-cirrhosis group at a 1:4 ratio using the SPSS random number table. Statistical analyses were performed using SPSS software (Version 25.0; SPSS Inc, Chicago, IL).

### Identification of decompensated cirrhosis, covariates, and outcomes

Decompensated cirrhosis was identified when the following clinical symptoms occurred concomitantly with cirrhosis: hepatic encephalopathy, variceal hemorrhage, hepatorenal syndrome, spontaneous bacterial peritonitis, hepatopulmonary syndrome, and/or ascites [14]. For the cirrhosis group, data regarding the etiology of cirrhosis, treatment measures for cirrhosis, and examination indicators related to cirrhosis were collected. Covariates included maternal age, body mass index (BMI), twin pregnancy, breech presentation, prior cesarean section, parity, pregestational diabetes mellitus, and chronic hypertension [8, 15].

Maternal and neonatal indicators were compared between the cirrhosis group and non-cirrhosis group. Obstetric outcomes included cesarean section, induction of labor (artificially stimulating the uterus to initiate labor), postpartum hemorrhage (defined as blood loss ≥ 500 ml at vaginal delivery or blood loss ≥ 1,000 ml at cesarean section), preeclampsia, gestational diabetes, placental abruption, oligohydramnios, intrahepatic cholestasis of pregnancy (ICP; defined as itching in skin of normal appearance with a raised peak while detecting total random bile acid concentrations of 10 µM/L or more), thrombocytopenia (a platelet count below 150 × 10^9^/L), maternal death, fetal distress (fetus’s acute and chronic hypoxic symptoms under the influence of various uterine factors), transfusion, and peripartum maternal intensive care unit (ICU) admission. Neonatal outcomes included fetal or neonatal demise, preterm birth (giving birth at or after 28 0/7 weeks of gestation and before 37 0/7 weeks of gestation), small for gestational age (SGA; defined as a birthweight below the tenth centile for gestational age), and an Apgar score of less than 7 at 5 min.

### Variable assignment

To investigate their associations with preterm birth in women with cirrhosis, we assigned values to intrahepatic cholestasis of pregnancy (ICP) and decompensated cirrhosis. The assignment for ICP (Without = 0, With = 1). The assignment for decompensated cirrhosis (Without = 0, With = 1).

### Statistical analysis

All data analyses were performed using the statistical software package SPSS version 25.0. Clinical characteristics and pregnancy outcomes were compared using Student *t*-tests and Mann–Whitney tests for continuous variables, as well as Fisher’s exact test or *χ*^*2*^ test for categorical variables. Logistic regression was performed to calculate obstetric outcomes among women with cirrhosis compared to those without cirrhosis. The results are presented as the odds ratio (OR) with 95% confidence intervals. All tests were two-tailed, and a *P*-value < 0.05 was considered statistically significant when comparing the cirrhosis group and the non-cirrhosis group.

A forward stepwise logistic regression model was designed to find the most significant variables predicting the rate of preterm birth among pregnant women with cirrhosis. Independent factors associated with the outcome in the univariate analysis (*P* < 0.1) were included in a multivariate regression analysis to predict the occurrence of preterm birth in the model-development dataset. The screening performance of the prediction model was assessed using the receiver operating characteristic (ROC) curve, and the area under curve (AUC).

This study was conducted according to the guidelines of the Declaration of Helsinki, and approved by the Ethics Committee of Third Affiliated Hospital of Sun Yat-sen University (No. II2023–053-01). The requirement for informed consent was waived by the Ethics Committee of Third Affiliated Hospital of Sun Yat-sen University because of the retrospective nature of the study.

## Results

### Characteristics of pregnant women in the cirrhosis group

The incidence of cirrhosis in pregnancy was 0.06% (50/81, 554). Characteristics of pregnant women in the cirrhosis group are presented in Table 1. The most common etiology was viral hepatitis B (53.49%), whereas no cases of alcoholic liver disease were reported among pregnant women in the cirrhosis group. Decompensated cirrhosis was identified in 13 pregnant women in our study. Only 17 patients underwent endoscopy either before or during pregnancy, and 14 out of 17 patients were found to have varices. Of the 17 patients, 10 underwent endoscopy during pregnancy, 4 were examined in the second trimester, and 6 were examined in the third trimester. No immediate risk to the mother or fetus was observed in the patients who underwent endoscopy during pregnancy. There were no cases of variceal hemorrhage nor maternal death during the peripartum period; however, six pregnant women were admitted to the general obstetric ward during pregnancy and then were transferred to the ICU after delivery.

**Table 1:** Characteristics of pregnant women in the cirrhosis group (*n* = 43)

Variable	*n* (%)
**Etiology**	
Hepatitis B	23 (53.49%)
Hepatitis C	3 (6.98%)
Autoimmune hepatitis	2 (4.65%)
Budd–Chiari syndrome	4 (9.30%)
Wilson's disease	3 (6.98%)
Primary biliary cirrhosis	1 (2.33%)
Cryptogenic cirrhosis	5 (11.63%)
Drug-induced hepatitis	1 (2.33%)
Parasitic infection	1 (2.33%)
**Compensatory**	
Compensated cirrhosis	30 (69.77%)
Decompensated cirrhosis	13 (30.23%)
**Maternal death**	0 (0)
**Intensive Care Unit**	6 (13.95%)

### Characteristics of pregnant women with decompensated cirrhosis

The gestation at time of birth occurring in pregnant women with decompensated cirrhosis was 33.38 ± 3.78 weeks. The number of preterm births in the pregnant women with decompensated cirrhosis was 9. All 13 cases of decompensated cirrhosis were cesarean section deliveries. One of these women had spontaneous onset but refused vaginal delivery. None of the pregnant women with decompensation cirrhosis delivered with labor induction.

### Baseline characteristics of women in the cirrhosis group compared with those in the non-cirrhosis group

There were no significant differences between the two groups in terms of age, BMI, twin pregnancy, breech presentation, prior cesarean section, nulliparous, multiparous, pregestational diabetes, or chronic hypertension (Table 2).

**Table 2:** Baseline characteristics of pregnant women in the cirrhosis group and non-cirrhosis group

Characteristics	Cirrhosis group *n* = 43	Non-cirrhosis group *n* = 172	*P*
Age, mean (SD), y	31.88 ± 5.82	30.93 ± 4.03	0.314
BMI, mean (SD), kg/m^2^	20.19 ± 2.10	20.92 ± 2.47	0.073
Twin pregnancy	1(2.33%)	3(1.74%)	1
Breech presentation	3(6.98%)	7(4.07%)	0.686
Prior cesarean section	3(6.98%)	32(18.60%)	0.065
Nulliparous	24(55.81%)	70(40.70%)	0.074
Multiparous	19(44.19%)	102(59.30%)	0.074
Pregestational diabetes	2(4.65%)	3(1.74%)	0.572
Chronic hypertension	0(0.00%)	0(0.00%)	-

Data are expressed as mean ± SD or *n* (%)

*SD* standard deviation

### Pregnancy outcomes of women in the cirrhosis group and non-cirrhosis group

In comparison to pregnant women without cirrhosis, pregnant women with cirrhosis displayed a higher risk of various adverse outcomes, including cesarean section, preterm birth, ICP, thrombocytopenia, and postpartum hemorrhage (*p* < 0.05; Table 3). None of the pregnant women in the cirrhosis group underwent induction of labor.

**Table 3:** Pregnancy outcomes of women in the cirrhosis group and non-cirrhosis group

Complication	Cirrhosis group (*n* = 43)	Non-cirrhosis group (*n* = 172)	OR (95% CI)	*P*
Cesarean section	36(83.72%)	62(36.05%)	9.12(3.83–21.72)	< 0.001*
Emergency caesarean	26(60.47%)	19(11.05%)	12.32(5.67–26.74)	< 0.001*
Elective caesarean	10(23.26%)	43(25%)	0.91(0.41–2.00)	0.812
Induction of labor	0(0.00%)	35(20.35%)	-	-
Postpartum hemorrhage	6(13.95%)	2(1.16%)	13.78(2.68–71.01)	0.002*
Pre-eclampsia	0(0.00%)	4(2.33%)	-	-
GDM	8(18.60%)	19(11.05%)	1.84 (0.75–4.55)	0.186
Placental abruption	2(4.65%)	0(0.00%)	-	-
Oligohydramnios	1(2.33%)	11(6.40%)	0.35 (0.04–2.78)	0.319
ICP	18(41.86%)	6(3.49%)	19.92 (7.22–54.97)	< 0.001*
Thrombocytopenia	28(65.12%)	12(6.98%)	24.89 (10.55–58.74)	< 0.001*
Maternal death	0(0.00%)	0(0.00%)	-	-
Fetal distress	2(4.65%)	12(6.98%)	0.650(0.14–3.02)	0.583
Transfusion	8(18.60%)	0(0.00%)	-	-
ICU	6(13.95%)	0(0.00%)	-	-
Preterm Birth	20(46.51%)	6(3.49%)	24.06 (8.75–66.13)	< 0.001*
Emergency caesarean	17(39.53%)	4(2.33%)	-	-
Elective caesarean	1(2.33%)	0(0.00%)	-	-
Induction of labor	0(0.00%)	0(0.00%)	-	-
Spontaneous onset	2(4.65%)	2(1.16%)	-	-
SGA	3(6.98%)	13(7.56%)	0.92 (0.25–3.37)	0.897
Apgar score ≤ 7 at 5 min	3(6.82%)	0(0.00%)	-	-

*OR* Odds ratio, *CI* Confidence interval, *GDM* Gestational diabetes mellitus, *ICP* Intrahepatic cholestasis of pregnancy, *ICU* Intensive care unit, *SGA* Small for gestational age

*, *P*-value < 0.05 indicates a significant difference

Twenty pregnant women in the cirrhosis group experienced preterm birth, leading to an incidence rate of 46.51% (20/43), which was significantly higher than that of the non-cirrhosis group. The gestation at time of preterm birth in the cirrhosis group was significantly smaller than that in the non-cirrhosis group (32.25 ± 2.81 weeks versus 34.83 ± 1.47 weeks). Of the twenty pregnant women, 17 were emergency cesarean section; 1 was an elective caesarean section; and 2 were spontaneous onset. No preterm birth occurred as a result of iatrogenic induction of labor. Reasons for emergency cesarean section in patients with preterm birth included fetal distress (2), elevated bile acids (6), placental abruption (1), placenta previa with hemorrhage (2), spontaneous onset with breech presentation (1), spontaneous onset but refused vaginal delivery (1) and decompensated cirrhosis (4). Most of the emergency caesarean sections were due to obstetric indications.

### Univariate logistic regression analysis of influencing factors for preterm birth in pregnant women with liver cirrhosis

Univariate logistic regression analysis was carried out on the dependent variables of the outcome of preterm birth and the independent variables listed in Table 4. The regression coefficient β for variables PT, TBIL, ICP, and decompensated cirrhosis were positive, and the OR values were greater than 1, indicating that prothrombin time (PT), total bilirubin (TBIL), ICP, and decompensated cirrhosis were risk factors for the prognosis of preterm birth (*p* < 0.05; Table 4).

**Table 4:** Univariate logistic regression analysis of influencing factors for preterm birth in pregnant women with liver cirrhosis

Variables	β	SE	Wald	*p*	OR (95% CI)
Age (y)	− 0.016	0.053	0.091	0.763	0.98 (0.89–1.09)
Urea (mmol/L)	0.073	0.268	0.074	0.786	1.08 (0.64–1.82)
Cre (µmol/L)	0.025	0.027	0.820	0.365	1.03 (0.97–1.08)
INR	4.860	2.711	3.213	0.073	129.06 (0.64–26,229.58)
PT (sec)	0.551	0.254	4.707	0.03	1.73 (1.06–2.85)
Fib (g/L)	− 0.517	0.270	3.670	0.055	0.60 (0.35–1.01)
TBIL (µmol/L)	0.126	0.551	6.080	0.014	1.13 (1.03–1.25)
ICP	2.405	0.735	10.701	0.001	11.08 (2.62–46.84)
PLT (× 10^9^/L)	0.003	0.004	0.625	0.429	1.00 (0.99–1.01)
Hb (g/L)	− 0.014	0.018	0.597	0.440	0.99 (0.95–1.02)
Decompensated cirrhosis	2.686	1.120	5.752	0.016	14.67 (1.63–131.66)

*SE* Standard error, *OR* Odds ratio, *Cre* Creatinine, *INR* International normalized ratio, *PT* Prothrombin time, *Fib* Fibrinogen, *TBIL* Total bilirubin, *ICP* Intrahepatic cholestasis of pregnancy, *PLT* Platelet, *Hb* Hemoglobin

### Multivariate logistic regression analysis of influencing factors for preterm birth in pregnant women with liver cirrhosis

As shown in Table 5, the regression equation used was as follows:

**Table 5:** Multivariate logistic regression analysis of influencing factors for preterm birth in pregnant women with liver cirrhosis

Variables	β	SE	Wald	*p*	OR (95%CI)
TBIL (µmol/L)	0.103	0.051	4.157	0.041	1.11 (1.00–1.23)
ICP	2.227	0.794	7.878	0.005	9.28 (1.96–43.94)
Constant	− 2.672	0.900	8.822	0.003	0.07

*ICP* Intrahepatic cholestasis of pregnancy, *OR* Odds ratio, *SE* Standard error, *TBIL* Total bilirubin

$$Logit\left(P\right)= -2.672+0.103 \times TBIL+ 2.227 \times ICP$$

$$P =1/1+{e}^{-(-2.672+0.103\times TBIL+2.227\times ICP)}$$

### Prediction accuracy rate of the model

Of the 43 pregnant women with cirrhosis, the model predicted 20 women had preterm births and 23 women had term births. Of the 20 preterm births predicted by the model, 4 were not preterm births, while 4 of the 23 term births predicted by the model were preterm births, producing a model prediction accuracy rate of 81.4%.

### ROC curve for predicting probability of preterm birth in pregnant women with cirrhosis

**Fig. 1:**
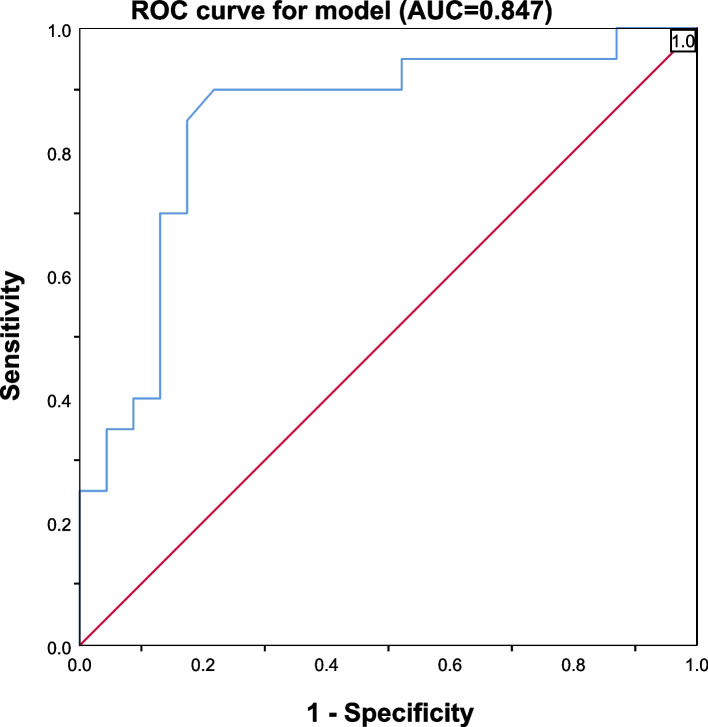
Receiver operating characteristic (ROC) curve for the multivariable logistic regression model used to predict preterm birth in pregnant women with cirrhosis. The area under the ROC curve (AUC) was 0.847 (95%CI: 0.723–0.971)

Using the above-mentioned regression equation, we obtained probability predictions for preterm birth for the group of 43 pregnant women with cirrhosis. We then plotted the ROC curve, which demonstrated an area under the ROC curve (AUC) of 0.847 (95% CI: 0.723–0.971). The point closest to the upper left of the curve exhibited a sensitivity of 90% and specificity of 78%. The Youden Index was calculated as follows: Youden Index = Sensitivity + Specificity − 1. The cutoff point was 0.31, when the Youden Index was at its maximum (Fig. 1).

## Discussion

In this study, the incidence of cirrhosis during pregnancy was 50 per 81,554 pregnancies, which was higher than previously reported [16, 17]. Viral hepatitis was identified as the main etiological cause of cirrhosis in pregnant women. None of the pregnant women with cirrhosis died during pregnancy. Cirrhosis in pregnancy was associated with increased risk of cesarean section, preterm birth, ICP, thrombocytopenia, and postpartum hemorrhage. The incidence rate of preterm birth was significantly higher in pregnant women with cirrhosis compared to pregnant women without cirrhosis. The regression model provides preliminary evidence for factors associated with preterm birth in pregnant women with cirrhosis in China, where viral hepatitis has been identified as the main etiological cause of cirrhosis.

In our study, Pregnant women with viral hepatitis account for 60% of all cirrhosis patients. Hepatitis B accounts for the vast majority of patients with viral hepatitis. This differs from previous studies performed in other countries, where alcoholic liver disease or nonalcoholic fatty liver disease was reported as the primary cause of liver cirrhosis during pregnancy [10, 17, 18]. The most common cause of cirrhosis in pregnancy is inconsistent worldwide. The etiology of liver cirrhosis varies according to national economic status. Chronic hepatitis B virus infection is common in China compare to other countries.

Earlier reports demonstrated a maternal mortality rate as high as 10.5% in pregnant women with cirrhosis [19]. However, with improved medical therapy and management of pregnant women with cirrhosis, it is likely that maternal mortality rates have decreased. Recent studies have shown that the mortality rate for pregnancies complicated by cirrhosis is rare, at less than 2% [8, 20]. Although previous studies have reported a statistical association between cirrhosis and maternal mortality, these events were fortunately rare. In our study, none of the women with cirrhosis died during the peripartum period. Mortality among pregnant women with cirrhosis was not as high in this study as was reported in previous studies. Cirrhosis is not an absolute contraindication to pregnancy; a more supportive position can be assumed for women with liver cirrhosis who wish to become pregnant.

Previous researches reported the most common cause of maternal mortality was variceal hemorrhage [21]. In our study, four patients in the cirrhosis group had a history of variceal bleeding before pregnancy, but none encountered variceal bleeding during the peripartum period. Only 39.53% patents in cirrhosis group underwent endoscopy either before or during pregnancy. Varices presented in most of the patients who underwent endoscopy. In our study, not all pregnant women with cirrhosis underwent an endoscopy. We do not know whether the remaining patients with cirrhosis who did not undergo endoscopy developed varices. Clinicians often recommend endoscopy for patients with decompensated cirrhosis or severe clinical manifestations, which might be a reason for the high rate of varices in patients who underwent endoscopy in our study. Previous studies have shown that endoscopy can be safely performed with minimal immediate risk to the mother and fetus during pregnancy. [22, 23]. In our study, no immediate risk to the mother or fetus was observed in the cases that underwent endoscopy during pregnancy. Endoscopy may be performed during pregnancy when strong indications are present.

Previous studies reported a wide range of cesarean section rates (25.6% to 81.4%) [8, 21, 24]. The incidence of cesarean section of 25.6% was reported in a study by Danielsson Borssén et al.[24]*.* However, this study included only 33 pregnant women with cirrhosis, all of whom had cirrhosis due to autoimmune hepatitis. In 2013, Rasheed et al*.* reported that the cesarean section rates of pregnancy outcome among patients with post-hepatitis live cirrhosis was 81.4%. In a study by Rasheed et al*.* [21], the decompensation rate in pregnant women with cirrhosis was as high as 63.6%. In 2018, Hagström et al. reported that cirrhosis was associated with an increased risk of cesarean section, with a cesarean section rate of 36% [8]. The main etiological causes of cirrhosis in Hagström’s study were viral hepatitis and autoimmune liver diseases. The proportion of patients with decompensated cirrhosis was not mentioned in Hagström’s study. Factors such as different causes of cirrhosis and different severity levels of cirrhosis may be the reasons for the differences in cesarean section rates reported by different studies. Patient/institutional preference and temporal trends in obstetric practice may be another reason explaining these discrepancies.

In our study, cesarean section was more common in the cirrhosis group than in the non-cirrhosis group. The caesarean section rate was 83.72% in pregnant women in the cirrhosis group. Most of the caesarean sections were emergency operations performed due to obstetric indications. In our study, none of the pregnant women in the cirrhosis group underwent an induction of labor. Obstetricians have expanded the indications for cesarean section for various reasons. One important reason could be the concern that the force of breath-holding during vaginal delivery may lead to gastroesophageal variceal bleeding [25, 26]. Another reason could be the low rate of labor inductions. Pregnant women with cirrhosis often suffer from obstetric problems while the fetus is not at term. At this time, the cervical maturity is poor, and the success rate of labor induction is low. Emergency cesarean section can improve the poor condition of mother and fetus more quickly than can labor induction. These may be the reasons that pregnant women were given an emergency cesarean section instead of labor induction in our institution. It is important to note that pregnancy with cirrhosis alone is not an indication for cesarean section. There is insufficient evidence for the ideal delivery mode of pregnancy with cirrhosis.

We observed an increased risk of ICP in pregnant women with cirrhosis. This phenomenon has received little attention in previous studies. However, a recent study by Flemming et al. suggested an association between cirrhosis and ICP [9], which aligns with our findings. ICP is associated with severe adverse pregnancy outcomes, such as preterm birth, fetal distress, and stillbirth [27]. Hence, pregnant women with cirrhosis need enhanced monitoring of total bile acid levels to discover potential complications related to ICP.

Prior studies reported that that women with cirrhosis have higher rates of postpartum hemorrhage [16, 21]. Cirrhosis is often accompanied by splenomegaly and hypersplenism, resulting in thrombocytopenia [28]. We observed a 13.78-fold increased risk of postpartum hemorrhage in the cirrhosis group compared to the non-cirrhosis group, which may be attributed to the higher rates of cesarean section and thrombocytopenia among women with cirrhosis. However, due to the small sample size in our study, the result has a wide-confidence interval. Pregnant women with cirrhosis may suffer a higher risk of postpartum hemorrhage. Consideration for additional measures to prevent such complications can be made until further research is available.

Preterm birth in pregnant women with cirrhosis is common. In our study, Preterm births were iatrogenic in most cases. Pregnancy with cirrhosis did not appear to increase the risk of spontaneous onset preterm delivery.

To address the issue of accurate prediction of preterm birth in pregnant women with cirrhosis, we developed a prediction model using logistic regression analysis. The factors used included PT, TBIL, ICP, and decompensated cirrhosis. Finally, TBIL and ICP were included in the regression equation. These two factors are simple and easy to obtain, which makes them convenient for clinicians to use.

Decompensated cirrhosis would theoretically increase the risk of complications when continuing the pregnancy. However, in our study, the statistical analyses showed that decompensated cirrhosis was not a significant variable for predicting preterm birth. Therefore, the variable of decompensated cirrhosis was not included in the regression model. One possible reason is that the sample size of our study was small. Another possible reason is that some pregnant women did not show obvious signs of decompensation cirrhosis until after reaching term.

The regression model showed a prediction accuracy of 81.4% and an area under the ROC curve of 0.847, indicating that these factors hold great value in predicting the possibility of preterm birth in pregnant women with cirrhosis. The regression model offers preliminary evidence for using these factors as key predictors of preterm birth, but more research is needed.

There are several limitations to our study, which was a single-site clinical study with a relatively small sample size. Furthermore, similar to prior researches [9, 16], we used ICD-10 code to screen for pregnant women with cirrhosis. The validity of using the ICD-10 code must be considered. The ICD code may have been misclassified due to incomplete or inaccurate reporting during the discharge diagnoses. A single hospital ICD code, although specific, may not be sensitive enough; some patients with early-stage cirrhosis may still be missed. In our study, ICD-10 code for cirrhosis before or during pregnancy, ICD-10 code for hepatic decompensation, and ICD-10 code for causes of cirrhosis were applied to reduce the potential for missing a case of liver cirrhosis. Due to the small sample size in our study, the 95% confidence intervals of some OR values were large. Another limitation is that the study was conducted in China, where the most common factor associated with cirrhosis is hepatitis, which is a different primary cause of cirrhosis than reported for other countries. Furthermore, different approaches in clinical practice might be an important influencing factor to consider. China has a lower induction rate compared to that of other countries. Cirrhosis due to other reasons (i.e., alcohol) and treatment of cirrhosis in other countries where there is liberal use of labor induction may yield much different results compared to the outcomes of this study. These findings therefore cannot be generalizable.

The major strength of our study is the development of a prediction model for the occurrence of preterm birth in pregnant women with cirrhosis. To the authors’ knowledge, this study is the first to establish such a model. Our findings highlight the significance of TBIL and ICP as risk factors for preterm birth in pregnant women with cirrhosis. We recognize that the performance of the proposed screening model may not be optimal, and further research with a large sample size is needed to verify the screening accuracy. In addition, we added the experience of managing pregnant women with cirrhosis.

## Conclusions

Our study reveals that cirrhosis in pregnancy is well tolerated, but it is accompanied by an increased risk of various complications, such as cesarean section, preterm birth, ICP, thrombocytopenia, and postpartum hemorrhage. Notably, the incidence of preterm birth is significantly higher in this population. The regression model established in our study shows high accuracy in predicting preterm birth in pregnant women with cirrhosis. We have demonstrated that high levels of maternal serum TBIL and the presence of ICP are associated with an increased risk of preterm birth in these individuals. Hence, it is crucial to detect the presence of ICP and assess the TBIL levels in pregnant women diagnosed with cirrhosis.

## Data Availability

The datasets used and/or analyzed during the current study are available from the corresponding author on reasonable request.

## Abbreviations

ROC: Receiver operating characteristic
AUC: Area under the ROC curve
ACG: American College of Gastroenterology
EASL: European Association for the Study of the Liver
BMI: Body mass index
ICP: Intrahepatic cholestasis of pregnancy
ICU: Intensive care unit
SGA: Small for gestational age
OR: Odds ratio
INR: International normalized ratio
PT: Prothrombin time
TBIL: Total bilirubin
MELD: Model for End-stage Liver Disease
CI: Confidence interval
GDM: Gestational diabetes mellitus
SE: Standard error
Cre: Creatinine
PT: Prothrombin time
Fib: Fibrinogen
PLT: Platelet
Hb: Hemoglobin
